# Ethyl 1,3-dimethyl-1*H*-indole-2-carboxyl­ate

**DOI:** 10.1107/S1600536808034156

**Published:** 2008-10-25

**Authors:** A. Marx, G. Chakkaravarthi, G. Gobirajeshwaran, A. K. Mohanakrishnan, V. Manivannan

**Affiliations:** a Department of Physics, Presidency College, Chennai 600 005, India; b Department of Physics, CPCL Polytechnic College, Chennai 600 068, India; c Department of Organic Chemistry, University of Madras, Guindy Campus, Chennai 600 025, India

## Abstract

In the title compound, C_13_H_15_NO_2_, the plane of the indole ring forms a dihedral angle of 5.26 (6)° with the ester group and the ethyl side-chain C atoms. The crystal packing is stabilized by weak inter­molecular C—H⋯O and C—H⋯π inter­actions.

## Related literature

For biological activities of indole derivatives, see: Okabe & Adachi (1998 ▶); Schollmeyer *et al.* (1995 ▶). For related structures, see: Chakkaravarthi *et al.* (2007 ▶, 2008 ▶).
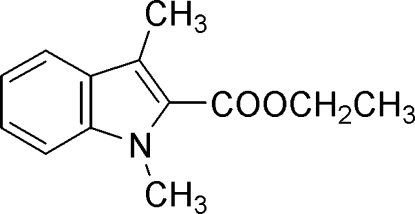


## Experimental

### 

#### Crystal data

C_13_H_15_NO_2_
*M* *_r_* = 217.26
Monoclinic, 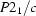

*a* = 7.5511 (3) Å
*b* = 12.2476 (6) Å
*c* = 12.9449 (5) Å
β = 105.488 (2)°
*V* = 1153.71 (9) Å^3^
*Z* = 4
Mo *K*α radiation
μ = 0.08 mm^−1^
*T* = 295 (2) K
0.25 × 0.20 × 0.20 mm

#### Data collection

Bruker Kappa APEXII diffractometer
Absorption correction: multi-scan (*SADABS*; Sheldrick, 1996 ▶) *T*_min_ = 0.979, *T*_max_ = 0.983
15440 measured reflections
3620 independent reflections
2068 reflections with *I* > 2σ(*I*)
*R*_int_ = 0.026

#### Refinement

*R*[*F* ^2^ > 2σ(*F* ^2^)] = 0.056
*wR*(*F* ^2^) = 0.184
*S* = 1.03
3620 reflections
148 parameters
H-atom parameters constrained
Δρ_max_ = 0.21 e Å^−3^
Δρ_min_ = −0.17 e Å^−3^

### 

Data collection: *APEX2* (Bruker, 2004 ▶); cell refinement: *SAINT*; data reduction: *SAINT*; program(s) used to solve structure: *SHELXS97* (Sheldrick, 2008 ▶); program(s) used to refine structure: *SHELXL97* (Sheldrick, 2008 ▶); molecular graphics: *PLATON* (Spek, 2003 ▶); software used to prepare material for publication: *SHELXL97*.

## Supplementary Material

Crystal structure: contains datablocks I, global. DOI: 10.1107/S1600536808034156/bq2100sup1.cif

Structure factors: contains datablocks I. DOI: 10.1107/S1600536808034156/bq2100Isup2.hkl

Additional supplementary materials:  crystallographic information; 3D view; checkCIF report

## Figures and Tables

**Table 1:** Hydrogen-bond geometry (Å, °)

*D*—H⋯*A*	*D*—H	H⋯*A*	*D*⋯*A*	*D*—H⋯*A*
C2—H2⋯O1^i^	0.93	2.53	3.401 (2)	156
C12—H12*A*⋯*Cg*1^ii^	0.97	2.76	3.646 (2)	152

Symmetry codes: (i) 
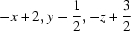
; (ii) 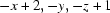. *Cg*1 is the centroid of the C1–C6 ring.

**Fig. 1.**
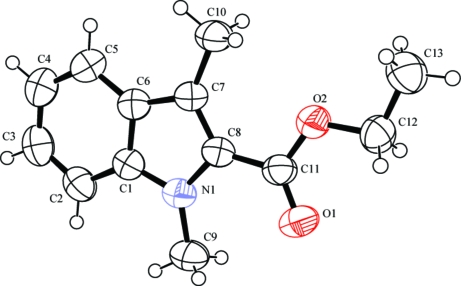
The molecular structure of (I), with atom labeling scheme. Displacement ellipsoids are drawn at 50% probability level.

**Fig. 2.**
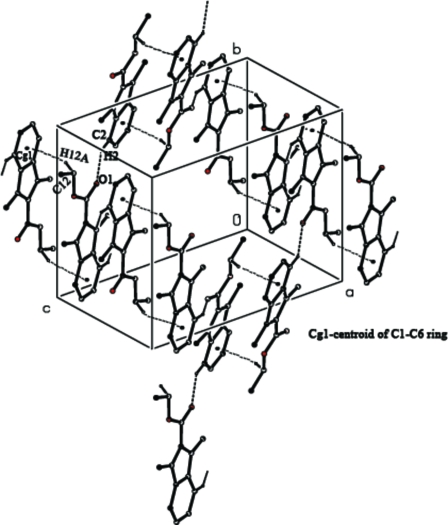
The crystal structure of (I), viewed down the a face. For the sake of clarity, H atoms not involved in interaction have been omitted.

## supplementary crystallographic information

### Comment

The indole derivatives are found to possess antibacterial (Okabe and Adachi, 1998) and antitumour activities (Schollmeyer *et al.*, 1995). In continuation to our studies in indole derivatives, we determine the crystal structure of the title compound (I). The geometric parameters of the molecule of (I) (Fig. 1) agree well with the reported structures (Chakkaravarthi *et al.*, 2007; Chakkaravarthi *et al.*, 2008).

The five- (N1/C1/C6/C7/C8) and six- (C1—C6) membered rings in the indane group are almost planar, with a dihedral angle of 1.67 (6)° between these rings. The plane of indole ring forms a dihedral angle of 5.26 (6)° with the ester group. The molecular packing is stabilized by weak intramolecular C—H···O interaction and the crystal packing of (I) is stabilized by weak intermolecular C—H···O and C—H···π interactions (Table 1) (Fig. 2).

### Experimental

To a stirred suspension of NaH (0.6 mmol, hexane washed) in THF (2 ml), a solution of 2-carbethoxy-3-methyl indole (0.5 mmol) in THF (2 ml) was added and stirred for 30 minutes at room temperature. To the reaction mixture, a solution of Iodomethane (0.6 mmol) was added and stirring was continued for further 6 hr. After the indole was consumed (monitored by TLC), the reaction mixture was quenched with cold dil HCl (25 ml), extracted with ethyl acetate (2 *x* 10 ml) and dried (Na_2_SO_4_). Removal of solvent followed by crystalization (hexane) afforded as yellow crystal.

### Refinement

H atoms were positioned geometrically and refined using riding model with C—H = 0.93 Å and *U*_iso_(H) = 1.2Ueq(C) for aromatic H atoms, C—H = 0.97 Å and *U*_iso_(H) = 1.2Ueq(C) for CH2 and C—H = 0.96 Å and *U*_iso_(H) = 1.5Ueq(C) for methyl H atoms.

### Crystal data

**Table:**

C_13_H_15_NO_2_	*F*(000) = 464
*M**_r_* = 217.26	*D*_x_ = 1.251 Mg m^−^^3^
Monoclinic, *P*2_1_/*c*	Mo *K*α radiation, λ = 0.71073 Å
Hall symbol: -P 2ybc	Cell parameters from 4312 reflections
*a* = 7.5511 (3) Å	θ = 2.3–24.1°
*b* = 12.2476 (6) Å	µ = 0.08 mm^−^^1^
*c* = 12.9449 (5) Å	*T* = 295 K
β = 105.488 (2)°	Block, yellow
*V* = 1153.71 (9) Å^3^	0.25 × 0.20 × 0.20 mm
*Z* = 4	

### Data collection

**Table:**

Bruker Kappa APEXII diffractometer	3620 independent reflections
Radiation source: fine-focus sealed tube	2068 reflections with *I* > 2σ(*I*)
graphite	*R*_int_ = 0.026
ω and φ scans	θ_max_ = 31.0°, θ_min_ = 2.3°
Absorption correction: multi-scan (SADABS; Sheldrick, 1996)	*h* = −10→10
*T*_min_ = 0.979, *T*_max_ = 0.983	*k* = −17→17
15440 measured reflections	*l* = −18→18

### Refinement

**Table:**

Refinement on *F*^2^	Primary atom site location: structure-invariant direct methods
Least-squares matrix: full	Secondary atom site location: difference Fourier map
*R*[*F*^2^ > 2σ(*F*^2^)] = 0.056	Hydrogen site location: inferred from neighbouring sites
*wR*(*F*^2^) = 0.184	H-atom parameters constrained
*S* = 1.03	*w* = 1/[σ^2^(*F*_o_^2^) + (0.0844*P*)^2^ + 0.1542*P*] where *P* = (*F*_o_^2^ + 2*F*_c_^2^)/3
3620 reflections	(Δ/σ)_max_ < 0.001
148 parameters	Δρ_max_ = 0.21 e Å^−^^3^
0 restraints	Δρ_min_ = −0.17 e Å^−^^3^

### Fractional atomic coordinates and isotropic or equivalent isotropic displacement parameters (Å^2^)

**Table:**

	*x*	*y*	*z*	*U*_iso_*/*U*_eq_	
C1	0.7616 (2)	−0.19859 (13)	0.57067 (13)	0.0527 (4)	
C2	0.7747 (2)	−0.30504 (16)	0.61130 (15)	0.0653 (5)	
H2	0.8324	−0.3194	0.6829	0.078*	
C3	0.7003 (3)	−0.38637 (16)	0.54252 (18)	0.0750 (5)	
H3	0.7075	−0.4577	0.5679	0.090*	
C4	0.6132 (3)	−0.36648 (16)	0.43493 (18)	0.0738 (5)	
H4	0.5646	−0.4246	0.3901	0.089*	
C5	0.5983 (2)	−0.26368 (16)	0.39465 (14)	0.0655 (4)	
H5	0.5386	−0.2510	0.3230	0.079*	
C6	0.67448 (19)	−0.17669 (13)	0.46276 (12)	0.0525 (4)	
C7	0.68784 (19)	−0.06336 (13)	0.44615 (11)	0.0501 (4)	
C8	0.78272 (19)	−0.01966 (13)	0.54312 (11)	0.0512 (4)	
C9	0.9305 (3)	−0.09425 (17)	0.73226 (14)	0.0757 (5)	
H9A	0.9384	−0.1651	0.7650	0.114*	
H9B	0.8689	−0.0449	0.7687	0.114*	
H9C	1.0519	−0.0675	0.7371	0.114*	
C10	0.6076 (2)	−0.00822 (15)	0.34166 (13)	0.0670 (5)	
H10A	0.5464	0.0575	0.3534	0.100*	
H10B	0.5209	−0.0561	0.2955	0.100*	
H10C	0.7038	0.0095	0.3088	0.100*	
C11	0.8333 (2)	0.09395 (14)	0.57069 (13)	0.0559 (4)	
C12	0.8335 (2)	0.27250 (13)	0.50367 (14)	0.0644 (4)	
H12A	0.9652	0.2837	0.5286	0.077*	
H12B	0.7771	0.3007	0.5573	0.077*	
C13	0.7583 (3)	0.32875 (18)	0.40024 (17)	0.0805 (6)	
H13A	0.8079	0.2962	0.3465	0.121*	
H13B	0.7913	0.4046	0.4077	0.121*	
H13C	0.6269	0.3219	0.3793	0.121*	
N1	0.82742 (18)	−0.10221 (11)	0.61980 (10)	0.0568 (4)	
O1	0.9033 (2)	0.12730 (12)	0.65918 (10)	0.0943 (5)	
O2	0.79272 (16)	0.15785 (9)	0.48555 (9)	0.0632 (3)	

### Atomic displacement parameters (Å^2^)

**Table:**

	*U*^11^	*U*^22^	*U*^33^	*U*^12^	*U*^13^	*U*^23^
C1	0.0489 (7)	0.0561 (9)	0.0535 (9)	0.0029 (6)	0.0146 (6)	0.0022 (7)
C2	0.0636 (9)	0.0662 (11)	0.0666 (11)	0.0026 (8)	0.0182 (8)	0.0136 (9)
C3	0.0774 (12)	0.0564 (11)	0.0939 (15)	−0.0017 (9)	0.0278 (11)	0.0091 (10)
C4	0.0742 (11)	0.0606 (11)	0.0849 (14)	−0.0079 (9)	0.0183 (10)	−0.0079 (10)
C5	0.0627 (9)	0.0696 (11)	0.0595 (10)	−0.0025 (8)	0.0081 (7)	−0.0072 (9)
C6	0.0476 (7)	0.0574 (9)	0.0519 (8)	0.0043 (6)	0.0122 (6)	0.0002 (7)
C7	0.0499 (7)	0.0552 (9)	0.0439 (8)	0.0047 (6)	0.0104 (6)	−0.0002 (6)
C8	0.0529 (8)	0.0556 (9)	0.0459 (8)	0.0041 (6)	0.0144 (6)	0.0012 (7)
C9	0.0883 (13)	0.0815 (13)	0.0488 (9)	0.0011 (10)	0.0035 (9)	0.0088 (9)
C10	0.0770 (11)	0.0675 (11)	0.0492 (9)	0.0038 (9)	0.0043 (8)	0.0050 (8)
C11	0.0624 (9)	0.0597 (10)	0.0462 (8)	0.0004 (7)	0.0155 (7)	−0.0025 (7)
C12	0.0722 (10)	0.0549 (10)	0.0682 (11)	0.0016 (8)	0.0226 (8)	−0.0044 (8)
C13	0.0902 (13)	0.0688 (12)	0.0804 (13)	0.0093 (10)	0.0189 (11)	0.0097 (10)
N1	0.0588 (7)	0.0631 (9)	0.0467 (7)	0.0022 (6)	0.0109 (6)	0.0036 (6)
O1	0.1481 (14)	0.0728 (9)	0.0498 (7)	−0.0162 (9)	0.0050 (8)	−0.0092 (6)
O2	0.0805 (8)	0.0518 (7)	0.0536 (7)	−0.0007 (5)	0.0113 (6)	−0.0014 (5)

### Geometric parameters (Å, °)

**Table:**

C1—N1	1.370 (2)	C9—N1	1.459 (2)
C1—C2	1.400 (2)	C9—H9A	0.9600
C1—C6	1.402 (2)	C9—H9B	0.9600
C2—C3	1.354 (3)	C9—H9C	0.9600
C2—H2	0.9300	C10—H10A	0.9600
C3—C4	1.394 (3)	C10—H10B	0.9600
C3—H3	0.9300	C10—H10C	0.9600
C4—C5	1.356 (3)	C11—O1	1.198 (2)
C4—H4	0.9300	C11—O2	1.319 (2)
C5—C6	1.404 (2)	C12—O2	1.443 (2)
C5—H5	0.9300	C12—C13	1.478 (3)
C6—C7	1.412 (2)	C12—H12A	0.9700
C7—C8	1.377 (2)	C12—H12B	0.9700
C7—C10	1.488 (2)	C13—H13A	0.9600
C8—N1	1.394 (2)	C13—H13B	0.9600
C8—C11	1.462 (2)	C13—H13C	0.9600
			
N1—C1—C2	130.38 (16)	H9A—C9—H9C	109.5
N1—C1—C6	108.26 (14)	H9B—C9—H9C	109.5
C2—C1—C6	121.33 (16)	C7—C10—H10A	109.5
C3—C2—C1	117.57 (17)	C7—C10—H10B	109.5
C3—C2—H2	121.2	H10A—C10—H10B	109.5
C1—C2—H2	121.2	C7—C10—H10C	109.5
C2—C3—C4	122.06 (18)	H10A—C10—H10C	109.5
C2—C3—H3	119.0	H10B—C10—H10C	109.5
C4—C3—H3	119.0	O1—C11—O2	122.77 (16)
C5—C4—C3	121.00 (18)	O1—C11—C8	125.30 (16)
C5—C4—H4	119.5	O2—C11—C8	111.93 (14)
C3—C4—H4	119.5	O2—C12—C13	107.02 (15)
C4—C5—C6	119.02 (17)	O2—C12—H12A	110.3
C4—C5—H5	120.5	C13—C12—H12A	110.3
C6—C5—H5	120.5	O2—C12—H12B	110.3
C1—C6—C5	119.01 (15)	C13—C12—H12B	110.3
C1—C6—C7	107.83 (14)	H12A—C12—H12B	108.6
C5—C6—C7	133.16 (15)	C12—C13—H13A	109.5
C8—C7—C6	106.51 (13)	C12—C13—H13B	109.5
C8—C7—C10	129.70 (16)	H13A—C13—H13B	109.5
C6—C7—C10	123.78 (14)	C12—C13—H13C	109.5
C7—C8—N1	109.60 (14)	H13A—C13—H13C	109.5
C7—C8—C11	129.14 (14)	H13B—C13—H13C	109.5
N1—C8—C11	121.25 (14)	C1—N1—C8	107.79 (13)
N1—C9—H9A	109.5	C1—N1—C9	123.47 (14)
N1—C9—H9B	109.5	C8—N1—C9	128.70 (15)
H9A—C9—H9B	109.5	C11—O2—C12	116.72 (13)
N1—C9—H9C	109.5		
			
N1—C1—C2—C3	−177.99 (16)	C6—C7—C8—C11	−179.58 (14)
C6—C1—C2—C3	0.1 (2)	C10—C7—C8—C11	−0.7 (3)
C1—C2—C3—C4	0.0 (3)	C7—C8—C11—O1	173.58 (17)
C2—C3—C4—C5	−0.5 (3)	N1—C8—C11—O1	−5.4 (3)
C3—C4—C5—C6	0.9 (3)	C7—C8—C11—O2	−6.4 (2)
N1—C1—C6—C5	178.77 (13)	N1—C8—C11—O2	174.65 (12)
C2—C1—C6—C5	0.3 (2)	C2—C1—N1—C8	178.04 (15)
N1—C1—C6—C7	−0.08 (16)	C6—C1—N1—C8	−0.24 (16)
C2—C1—C6—C7	−178.54 (14)	C2—C1—N1—C9	−0.1 (3)
C4—C5—C6—C1	−0.8 (2)	C6—C1—N1—C9	−178.40 (14)
C4—C5—C6—C7	177.72 (16)	C7—C8—N1—C1	0.48 (16)
C1—C6—C7—C8	0.37 (16)	C11—C8—N1—C1	179.62 (13)
C5—C6—C7—C8	−178.25 (16)	C7—C8—N1—C9	178.52 (15)
C1—C6—C7—C10	−178.61 (14)	C11—C8—N1—C9	−2.3 (2)
C5—C6—C7—C10	2.8 (3)	O1—C11—O2—C12	−1.2 (2)
C6—C7—C8—N1	−0.52 (15)	C8—C11—O2—C12	178.80 (13)
C10—C7—C8—N1	178.38 (15)	C13—C12—O2—C11	−173.84 (14)

### Hydrogen-bond geometry (Å, °)

**Table:**

*D*—H···*A*	*D*—H	H···*A*	*D*···*A*	*D*—H···*A*
C10—H10A···O2	0.96	2.49	2.858 (2)	103
C2—H2···O1^i^	0.93	2.53	3.401 (2)	156
C12—H12A···Cg1^ii^	0.97	2.76	3.646 (2)	152

Symmetry codes: (i) −*x*+2, *y*−1/2, −*z*+3/2; (ii) −*x*+2, −*y*, −*z*+1.
